# A Short-Term Feeding of Dietary Casein Increases Abundance of *Lactococcus lactis* and Upregulates Gene Expression Involving Obesity Prevention in Cecum of Young Rats Compared With Dietary Chicken Protein

**DOI:** 10.3389/fmicb.2019.02411

**Published:** 2019-10-25

**Authors:** Fan Zhao, Shangxin Song, Yafang Ma, Xinglian Xu, Guanghong Zhou, Chunbao Li

**Affiliations:** ^1^College of Food Science and Technology, Nanjing Agricultural University, Key Laboratory of Meat Products Processing, Ministry of Agriculture and Rural Affairs, Jiangsu Collaborative Innovation Center of Meat Production and Processing, Quality and Safety Control, Nanjing, China; ^2^School of Food Science, Nanjing Xiaozhuang University, Nanjing, China

**Keywords:** casein, chicken, *Lactococcus lactis*, AdipoQ, Irs1

## Abstract

Casein and chicken are assessed to contain high quality proteins, which are essential for human health. Studies have shown that ingestion of the two dietary proteins resulted in distinct effects on physiology, liver transcriptome and gut microbiota. However, its underlying mechanism is not fully understood, in particular for a crosstalk between gut microbiota and host under a specific diet intervention. We fed young rats with a casein or a chicken protein-based diet (CHPD) for 7 days, and characterized cecal microbiota composition and cecal gene expression. We found that a short-term intervention with a casein-based diet (CAD) induced a higher relative abundance of beneficial bacterium *Lactococcus lactis* as well as *Bifidobacterium pseudolongum*, which upregulated galactose metabolism of the microbiome compared with a CHPD. The CAD also upregulated gene expression involved in obesity associated pathways (e.g., Adipoq and Irs1) in cecal tissue of rats. These genes and the bacterial taxon were reported to play an important role in protecting development of obesity. Furthermore, the differentially represented bacterial taxon *L. lactis* was positively associated with these differentially expressed genes in the gut tissue. Our results provide a new insight into the crosstalk between gut microbiota and host in response to dietary proteins, indicating a potential mechanism of obesity prevention function by casein.

## Introduction

Meat and dairy products are the major dietary animal protein sources for human nutrition, which contain high quantities and balanced proportions of amino acids relative to human tissues (Li et al., 2011; FAO, 2013). Therefore, adequate consumption of such foods is essential for optimal growth, development, and human health (Wu, 2016). Chicken consumption has grown by 70% in developed countries since 1990 and it becomes one of the most widely consumed meat, indicating its increasing effects on human health. Casein is special for its high content of branched chain amino acids (BCAAs) (Rafiq et al., 2016), and has a potential to reduce body mass gain and diet-induced obesity (Lillefosse et al., 2013; Liisberg et al., 2016).

Although chicken protein and casein were assessed as high quality proteins, a short-term intervention from our group demonstrated that the two dietary proteins resulted in distinct physiological and liver transcriptomic changes in young rats (Song et al., 2016b). A number of studies have shown that there is a close association among obesity, high fat diet and gut microbiota (Martinez et al., 2017). However, several long-term animal studies have also shown that casein or CHPDs varied in their effects on obesity development in high fat diet fed mice (Liisberg et al., 2016) and gut microbiota in normal fat diet fed rats (Zhu et al., 2015), indicating a crucial impact of the protein sources. Recently, gut microbiota has been shown to play a critical role in human health by affecting physiology, energy homeostasis or immune system (Rooks and Garrett, 2016; Smidt et al., 2016; Gomes et al., 2018). Diet intake could determine the diversity and metabolic outputs of the microbial community (Zmora et al., 2019). Taken together, dietary protein-associated changes in the gut microbiota could be causally linked with host metabolism. However, the associations between gut microbiota and host in response to dietary proteins are less investigated. Also, most of the associated analyses on gut microbiota were conducted based on 16s rRNA sequencing, which may cause a bias.

In this study, we fed young rats with a casein or a CHPD for 7 days, and characterized cecal microbiota composition and gene expression in cecal tissue by using shotgun metagenomics and transcriptome sequencing. The associations among gut bacteria, gene expression in cecum tissue and physiological responses were discussed.

## Materials and Methods

### Diets

Protein diets were prepared by Jiangsu Xietong, Inc., according to the AIN-93G formulation (Reeves et al., 1993). Casein or chicken protein was included in the diets. To ensure consistency between the diets, most diet ingredients were purchased from Dyets Inc. (Bethlehem, PA, United States). Chicken protein was prepared as follows. Chicken *pectoralis major* muscle was cooked in a 72°C water bath till a center temperature of 70°C. The cooked meat was chilled and minced. Fat was removed in dichloromethane and methanol mixture (1: 2, v:v). Chicken meat powder was then passed through a 25 screen. The powder consists of proteins (>90%) and a small amount of mineral and other micronutrients. The detailed information of the diet formula was listed in Supplementary Table S1.

### Animal Feeding

The animal experiment has been previously described (Song et al., 2016b), and all the experimental protocols were approved by the Animal Care Committee of Nanjing Agricultural University. In brief, after a 1-week adaptation period, 4-week-old male Sprague-Dawley rats were fed either a casein-based or a CHPD (10 rats each group). After 7 days feeding, rats were anesthetized with ether inhalation. Cecal contents and tissues were obtained and snap-frozen separately in liquid nitrogen. Three of the 10 samples in each group were randomly selected for metagenomic sequencing (cecal contents) and transcriptome (cecal tissues) analyses.

### Metagenomic Sequencing

#### DNA Extraction and Sequencing

Genomic DNA was extracted according to the protocols of Zoetendal et al. (2006). DNA library construction was performed following the manufacturer’s instruction (Illumina Hiseq 2000). Paired-end DNA libraries was built and sequenced with 100 bp read length from each end under an Illumina Hiseq2000 platform by the standard pipelines.

#### Data Processing

Data filtration was done using in-house scripts according to MOCAT pipeline (Kultima et al., 2012). Adaptor contamination, low-quality reads, and host contaminating reads were removed from the raw sequencing reads sets. Finally, high-quality data were obtained for metagenomic analysis.

#### Species Composition and Abundance Analysis

Known bacterial sequences were extracted from an NT database, and then, filtered reads were mapped onto these sequences by SOAPaligner (version 2.21) (Li et al., 2009). Mapped reads were classified at different taxonomic levels (including phylum, class, order, family, genus, and species), and the corresponding abundance was summarized. Negative binomial distribution difference test (DEseq2, an R package) was applied for differential analysis of the bacteria between the two dietary groups.

#### Assembly and Gene Prediction

The filtered data were assembled by SOAPdenovo (Li et al., 2008) (Version 1.06^1^) and assembly results were optimized using an in-house program (BGI, Shenzhen). MetaGeneMark (version 2.10, default parameters^2^) software was used to predict open reading frames (ORFs) based on assembly results (Zhu et al., 2010). ORFs from all samples were combined without redundancy (processed by software cd-hit, 4.6.1^3^) (Li and Godzik, 2006) to obtain a gene catalog. Sequencing reads were annotated using KEGG Orthology group assignments (Version 59). A DESeq2 R package was applied for differential analysis of KEGG Orthology (KO) based on readcount data between the two dietary groups. Gene set enrichment analysis (GSEA) was applied to evaluate changes in gene expression related to biological processes (Subramanian et al., 2005). Gene sets were retrieved from the expert-curated KEGG pathway database^4^.

### Transcriptome Sequencing

Total RNA was extracted from cecal tissue by using Takara MiniBEST universal RNA extraction kit (Takara, Kusatsu, Japan). RNA degradation and contamination was monitored on 1% agarose gels. RNA purity was checked using a NanoPhotometer^®^ spectrophotometer (IMPLEN, Los Angeles, CA, United States). RNA concentration was measured using a Qubit^®^ RNA Assay Kit in Qubit^®^ 2.0 Fluorometer (Life Technologies, Carlsbad, CA, United States). RNA integrity was assessed using a RNA Nano 6000 Assay Kit of the Bioanalyzer 2100 system (Agilent Technologies, Santa Clara, CA, United States).

### Library Preparation for Transcriptome Sequencing

A total amount of 3 μg RNA per sample was used as input material for the RNA sample preparations. Sequencing libraries were generated using NEBNext^®^ Ultra^TM^ RNA Library Prep Kit for Illumina^®^ (NEB, United States) following manufacturer’s recommendations and index codes were added to attribute sequences to each sample. Briefly, mRNA was purified from total RNA using poly-T oligo-attached magnetic beads. Fragmentation was carried out using divalent cations under elevated temperature in NEBNext First Strand Synthesis Reaction Buffer (5X). First strand cDNA was synthesized using random hexamer primer and M-MuLV Reverse Transcriptase (RNase H^–^). Second strand cDNA synthesis was subsequently performed using DNA Polymerase I and RNase H^–^. Remaining overhangs were converted into blunt ends via exonuclease/polymerase activities. After adenylation of 3′ ends of DNA fragments, NEBNext Adaptor with hairpin loop structure were ligated to prepare for hybridization. In order to select cDNA fragments of preferentially 150∼200 bp in length, the library fragments were purified with AMPure XP system (Beckman Coulter, Beverly, United States). Then 3 μl USER Enzyme (NEB, United States) was used with size-selected, adaptor-ligated cDNA at 37°C for 15 min and then increased to 95°C and kept for 5 min. PCR was performed with Phusion High-Fidelity DNA polymerase, Universal PCR primers and Index (X) Primer. Finally, PCR products were purified (AMPure XP system) and library quality was assessed on the Agilent Bioanalyzer 2100 system (Agilent Technologies, United States).

### Clustering and Sequencing

The clustering of the index-coded samples was performed on a cBot Cluster Generation System using TruSeq PE Cluster Kit v3-cBot-HS (Illumia, United States) according to the manufacturer’s instructions. After cluster generation, the library preparations were sequenced on an Illumina Hiseq platform (Illumina, United States) and 125 bp/150 bp paired-end reads were generated.

### Quality Control

Raw data of fastq format were firstly processed through in-house perl scripts. Clean reads were obtained by removing reads containing adapter, ploy-N and low quality reads from raw data. Q20, Q30, and GC content in the clean data were calculated. All the downstream analyses were based on the clean data.

### Reads Mapping to the Reference Genome

Reference genome and gene model annotation files were downloaded from genome website directly. Index of the reference genome was built using Bowtie v2.2.3 (Langmead and Salzberg, 2012) and paired-end clean reads were aligned to the reference genome using the TopHat v2.0.12 (Trapnell et al., 2009).

### Differential Expression Analysis

Differential expression analysis was performed on the basis of the negative binomial distribution model using the DESeq2 R package (1.24.0). The resulting *P* values were adjusted using the Benjamini-Hochberg’s approach for controlling the false discovery rate (FDR). Genes with an adjusted *P* value < 0.05 were assigned as differentially expressed genes.

### Pathway Analysis

Gene set enrichment analysis was applied to evaluate changes in gene expression related to biological processes (Subramanian et al., 2005). GSEA has multiple advantages over analyses of individual genes (Abatangelo et al., 2009). Gene sets were retrieved from the expert-curated KEGG pathway database (see text footnote 4). Only gene sets consisting of 15–500 genes were taken into account. For each comparison, genes were ranked on their *t*-values that were calculated by the empirical Bayes method. Statistical significance of GSEA results was determined using 1,000 permutations. The Enrichment Map v3.2.0 plugin for Cytoscape v3.7.1 was used for visualization and interpretation of the GSEA results (Merico et al., 2010). Enrichment maps were generated with gene sets that passed conservative significance thresholds (*P* < 0.05, FDR < 0.25).

### Real-Time Quantitative PCR Validation

Total RNA was extracted from cecum tissue using a TaKaRa MiniBEST Universal RNA Extraction Kit (Takara) followed by reverse transcription into cDNA using PrimeScript^TM^ RT Master Mix (Perfect Real Time) (Takara, Japan). Quantitative real-time PCR was performed with Applied Biosystems^TM^ QuantStudio^TM^ 6 Flex Real-Time PCR System (Life Technologies, Waltham, MA, United States). Data analysis was according to 2^–ΔΔCt^ method (Livak and Schmittgen, 2001). The casein fed group was set as the control group. The primers for each specific genes were listed in Supplementary Table S2.

### The Correlation Analysis of Gut Microbiota and Host Gene Expression in Cecum

Correlation analysis was performed between gut microbiota and host gene expression using mixOmics R package (version 6.1.1^5^) (Rohart et al., 2017). Correlation coefficients were calculated between all the bacterial species and the top 250 differentially expressed genes.

## Results

### The Short-Term Effect of Dietary Protein on Cecal Microbiota

Bacterial DNA was extracted from cecal contents of rats fed casein and CHPD for 7 days. DNA was sequenced using metagenome sequencing, which yielded 18 gigabases (Gb) of high-quality data with an average of 3 Gb per sample (Supplementary Table S3). The K-mer frequency distribution analysis showed that the sequencing data were reliable (Supplementary Figure S1).

**FIGURE 1:**
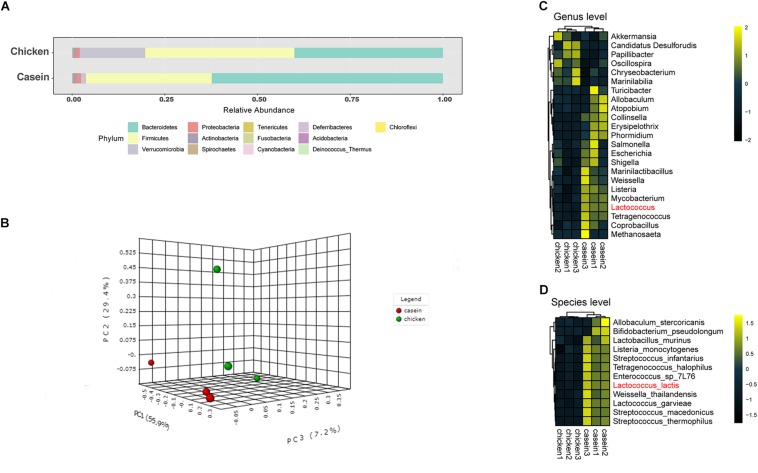
Cecal microbiota of the rats fed with casein and chicken protein-based diet. **(A)** The cecal microbial composition at the phylum level. **(B)** PCA scatter plot at the species level. **(C)** Differential bacteria at the genus level (*P* < 0.05). **(D)** Differential bacteria at the species level (*P*_adj_ < 0.1).

Great differences were observed in gut microbiota composition in cecum of rats fed with CAD and CHPD. At the phylum level, *Firmicutes* and *Bacteroidetes* accounted as average for 96.3 and 80.3% of cecal microbiota in CAD and CHPD groups (Figure 1A). Principle component analysis indicates a distinction of gut microbiota composition in CAD-fed rats from that of CHPD-fed rats (Figure 1B).

A negative binomial distribution model was applied to explore the differential microorganisms between the two groups. At the genus level, 23 bacteria showed significant difference with *P* values less than 0.05 (Figure 1C), whereas only the abundance of *Lactococcus* was found significantly higher in CAD group with an adjusted *P* value less than 0.05 (Supplementary Table S4). At the species level, 12 bacteria were significantly different. Among these species, *Lactococcus lactis* was the most variable microorganism between the two groups (*P*_adj_ < 0.05, Figure 1D, Supplementary Table S4).

**FIGURE 2:**
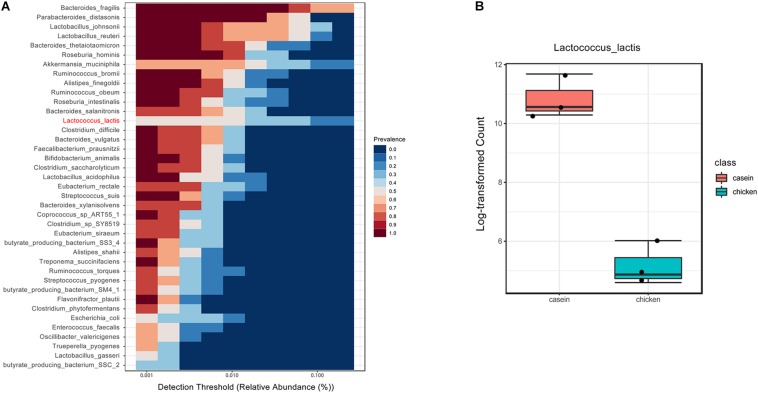
Core microbiota in cecum of rats fed with CAD and CHPD. **(A)** The bacteria prevalence at different detection thresholds by MicrobiomeAnalyst in which sample prevalence was set to 20% and relative abundance was set to 0.2%. The species marked in red was significantly different between the two diet groups. **(B)** The abundance of *Lactococcus lactis* in cecum of rats fed with CAD and CHPD.

Core microbiome analysis was performed at the species level using MicrobiomeAnalyst in which sample prevalence and relative abundance cut off value were set at 20 and 0.2%, respectively. Thirteen-nine species were identified as the core microbiome. *Bacteroides fragilis*, which belongs to genus *Bacteroides*, was the most dominant species among all the samples (Figure 2). The dominance of genus *Bacteroides* has been considered biomarkers of human diet and lifestyle (Gorvitovskaia et al., 2016). Of note, *L. lactis* was the only core species that was significantly different between the two diet groups.

**FIGURE 3:**
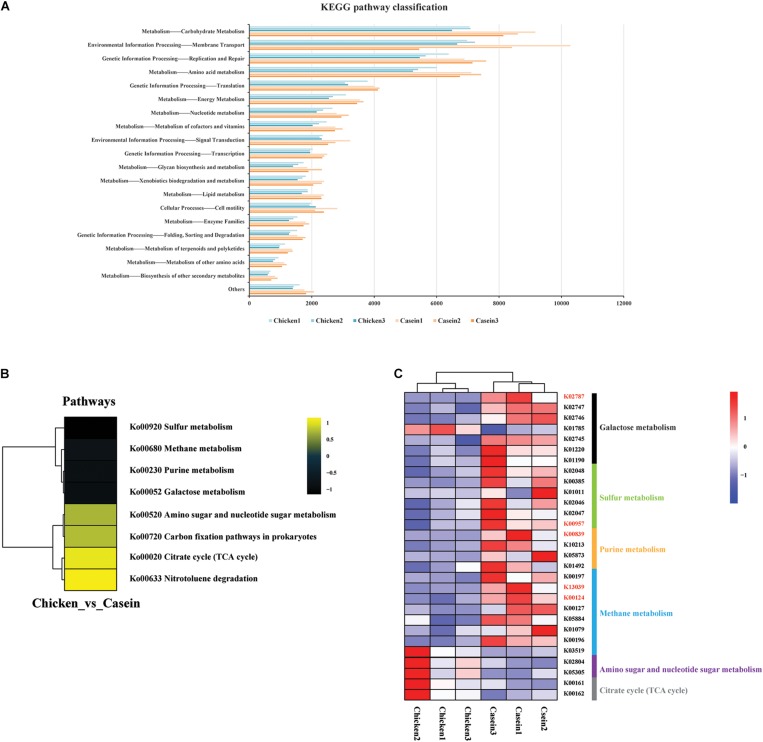
Functional analysis of the cecal microbiota of the rats fed chicken and casein protein diets. **(A)** KEGG pathway classification on level two. **(B)** Differential KEGG pathways analyzed by gage, R package. Only those with *P* value < 0.05 were shown. **(C)** KOs involved in the differential pathways between CAD and CHPD. Only the KOs with *P* value < 0.05 were shown. The KOs in red represent the *P*_adj_ < 0.05.

Kyoto Encyclopedia of Genes and Genomes analysis further revealed the alteration of pathways in gut microbiota in response to protein diets. Most of the mapped genes were involved in the carbohydrate metabolism, membrane transport, replication and repair, and amino acid metabolism (Figure 3A). A total of 19 KOs differed between CAD and CHPD, only two of which were significantly higher in CHPD group (Supplementary Table S5, *P*_adj_ < 0.05). The profile of enriched pathways was analyzed by GSEA to get the differential KEGG pathways in CAD and CHPD (Supplementary Table S6). CHPD showed more abundant microbial genomes involved in the citrate cycle pathway as compared to CAD, while CAD showed more abundant microbial genomes involved in sulfur, methane and galactose metabolism pathways (Figures 3B,C and Supplementary Figure S2; *P* < 0.05).

### The Short-Term Effect of Dietary Protein on Gene Expression of Rat Cecum

**FIGURE 4:**
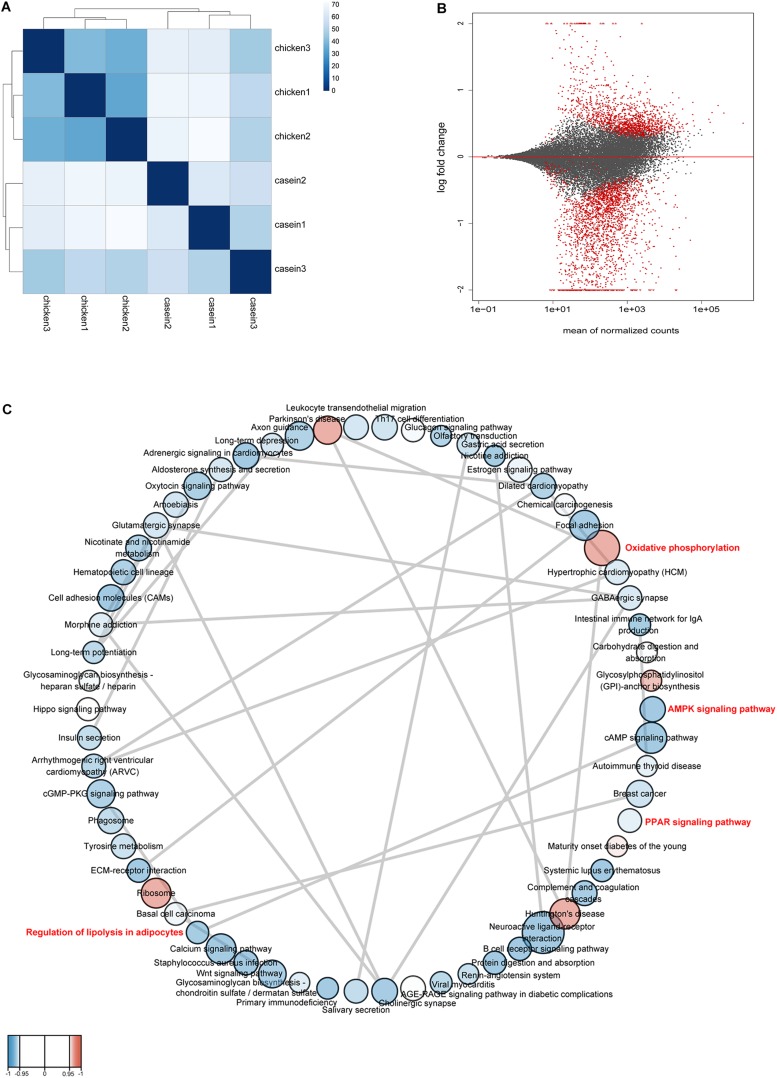
The profile of gene expression in rat cecum. **(A)** The distance between samples. Calculation was conducted by using variance stabilizing transformation method. **(B)** The MA-plot shows the log_2_ fold changes from the treatment over the mean of normalized counts. The red dots represent differentially expressed genes between the two groups. **(C)** Gene set network. The network was produced by Cytoscape v3.7.1 and Enrichment Map plugin v3.2.0. Nodes represent enriched gene sets (KEGG pathways) (*P* < 0.05, FDR < 0.25). The node size is proportional to the total number of genes within each pathway (from 15 to 500). The color of nodes indicates changes of pathways with red for up-regulation and blue for down-regulation in CHPD group compared with CAD group. The enrichment *P* value is mapped to the node color as a color gradient. The color changes from light to bright with the *P* value decreasing from 0.05 to 0. The gray lines between the round nodes represent overlap genes shared between the two pathways.

RNA was extracted from rat cecum tissue and was sequenced on Illumina HiSeq platform to identify differentially expressed genes. A total of 49.88 Gb high quality reads were obtained. The quality information of the data is shown in Supplementary Table S7. The samples from CAD and CHPD fed rats were well separated (Figure 4A). There were 2524 differential expressed genes between the two groups, of which 871 genes were upregulated in the CHPD group compared with the CAD group (Supplementary Table S8 and Figure 4B).

**FIGURE 5:**
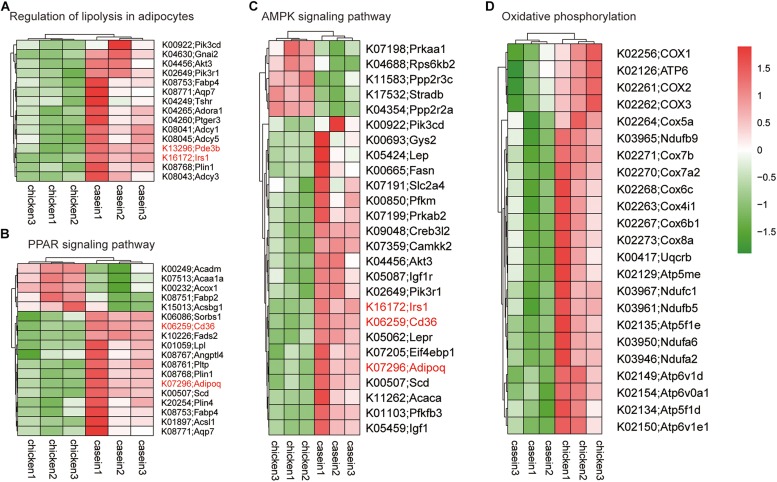
Heatmap of gene expression in differential KEGG pathways. The genes shown on the figure were differentially expressed (*P*_adj_ < 0.05) in cecum of rats fed CAD and CHPD. **(A)** Differentially expressed genes involved in Regulation of lipolysis in adipocytes. **(B)** Differentially expressed genes involved in PPAR signaling pathway. **(C)** Differentially expressed genes involved in AMPK signaling pathway. **(D)** Differentially expressed genes involved in oxidative phosphorylation.

A GSEA based on KEGG pathways was conducted to get better understanding of underlying biological processes induced by protein diets. We observed that 62 KEGG pathways were differentially regulated by diets (*P* < 0.05 and FDR < 0.25; Figure 4C and Supplementary Table S9). Six pathways were upregulated and 56 pathways were downregulated by CHPD compared with the CAD group. These pathways are mainly involved in regulation of obesity and lipolysis, including AMPK and PPAR signaling pathways. Adipoq (adiponectin, C1Q and collagen domain containing) and Irs1 (Insulin receptor substrate 1) may play a critical role in these signaling pathways. Most genes in AMPK and PPAR signaling pathways were downregulated by CHPD, while all differentially expressed genes involving oxidative phosphorylation were upregulated (Figure 5). This is in accordance with the published transcriptomic results in liver (Song et al., 2016b).

**FIGURE 6:**
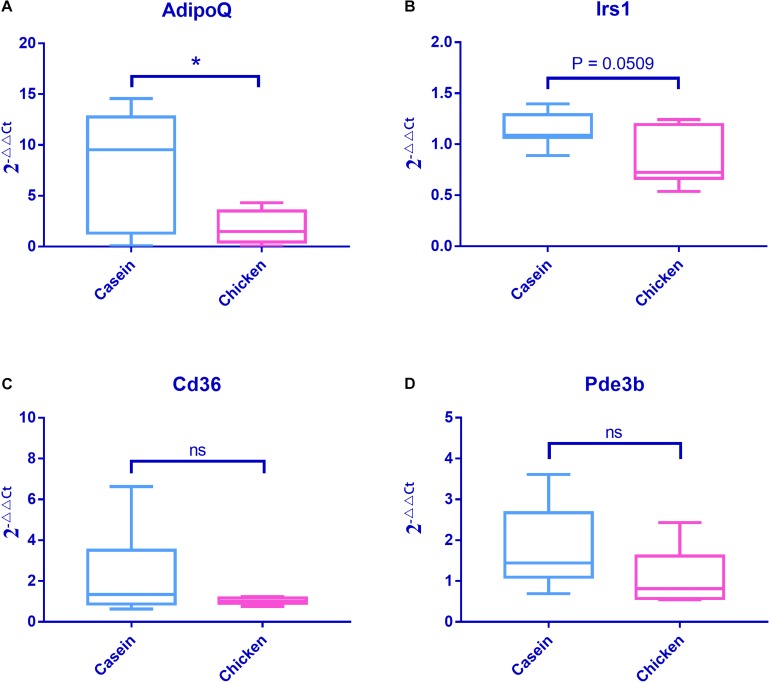
mRNA expressions of four obesity associated genes detected by Q-PCR. Fold changes were tested using Student’s *t* test. Casein was set as the control group when conducting the data analysis. **(A)** mRNA expression profile of AdipoQ. **(B)** mRNA expression profile of Irs1. **(C)** mRNA expression profile of Cd36. **(D)** mRNA expression profile of Pde3b.

Q-PCR analyses of Adipoq, Irs1, Cd36, and Pde3b genes confirmed the results of RNA sequencing. In particular, the Adipoq gene was more highly expressed in CAD than in CHPD group (*P* < 0.05, Figure 6).

### The Correlation Analysis of Cecal Bacteria and Gene Expression

**FIGURE 7:**
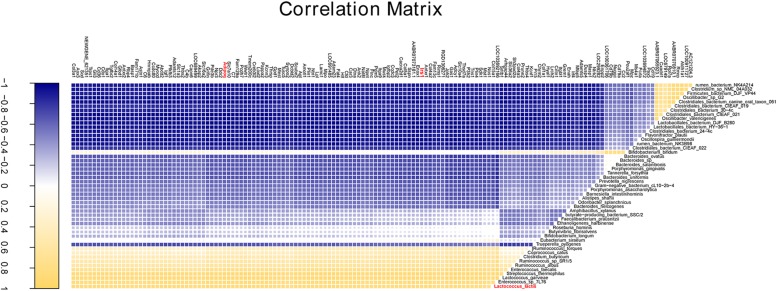
The correlation analysis of cecal bacteria and gene expression. The correlation profile between all annotated species and the top 250 variable genes with a coefficient value more than 0.85.

Partial least squares regression was applied for correlation analysis between cecal microbiota and gene expression of cecum tissue. Forty-nine species were found to be correlated with 148 of the top 250 most variable genes (correlation coefficients were greater than 0.85, Supplementary Table S10). Interestingly, the core variable species *L. lactis* was positively correlated with those genes such as AdipoQ, Irs1, Cd 36, and Pde3b (Figure 7) involved in obesity associated pathways.

## Discussion

The cecum is a major site for microbial fermentation of dietary components. It also plays a critical role in maintaining intestinal health (Brown et al., 2018). Here we reported a comprehensive comparison of the short-term diet effect at a normal dose on the cecal microbiota and gene expression in cecum tissue of young rats. This work relates to several previous studies on the effects of casein and CHPD on the growth performance, lipid metabolism and gut microbiota (Zhu et al., 2015; Liisberg et al., 2016; Song et al., 2016a, b).

However, it is little known how the gut microbiota and gene expression of gut tissue respond to a short-term intervention of dietary proteins. The samples used in the present study were obtained from the same animals as previous studies that focused on the effects of dietary protein sources on the physiology and liver metabolism (Song et al., 2016a,b,c).

Here, the composition of gut microbiota in cecum of CHPD-fed rats was found to be different from that of CAD-fed rats after a short-term diet intervention. In practice, diet may alter the gut microbiota within 2-day intervention (Biddinger et al., 2014; Zhao et al., 2017; Zmora et al., 2019).

At the genus level, the abundance of *Lactococcus* was higher in the CAD group. At the species level, *B. fragilis* and *Parabacteroides distasonis* were the top core species. *B. fragilis* is an important obligate anaerobe that colonizes the mammalian lower intestine (Moore and Holdeman, 1974). *P. distasonis* was initially classified to the genus *Bacteroides* but reclassified to genus *Parabacteroides* (Sakamoto and Benn, 2006), indicating a high similarity between the two species. This could be the reason why they acted similarly as the top core species in the present study.

*Lactococcus lactis* was also identified as a core species, which had a significantly higher abundance in the CAD group. However, in a relatively long term intervening study, we did not characterize the species *L. lactis* as an indicator for the CAD group (Zhu et al., 2015). This inconsistence could be due to different sequencing methods. A shotgun metagenomic sequencing was applied in the present study to get the information of metagenomic DNA without amplification, while 16S rRNA sequencing contained amplification step, which might cause a bias. Animal age and feeding period may be also the causes for such an inconsistence. In the previous study, 4-week-old Sprague-Dawley rats were used, which received a 7-day chow diet for acclimatization and then were subjected to 3-month diet intervention. In the present study, 3-week-old Sprague-Dawley rats were used and received 7-day chow diet for acclimatization and another 7-day diet intervention. The sampling time points were 18 weeks old for the previous study and 5 weeks old for the present study.

Kyoto Encyclopedia of Genes and Genomes analysis revealed the difference in metabolism functions of the cecal microbiota of rats between CAD and CHPD groups. Genes annotated to sulfur metabolism in CAD group were more abundant than those in CHPD group (Figure 3C), which could be attributed to higher content of sulfur-containing amino acid in casein (Supplementary Table S1). We also found that galactose metabolism was upregulated by CAD. This could be associated with higher abundance of *L. lactis*, which was reported to utilize lactose (Laroute et al., 2017). And the annotated gene *lacE* (encoding lactose-specific enzyme II of the PTS system, K02787) involving in galactose metabolism widely exists in *L. lactis* substrains (Passerini et al., 2010; Laroute et al., 2017). Although, lactose does not exist in casein, there are some glycoproteins (Gal and GalNAc) in kappacasein that might induce such kind of metabolism (Jollès and Fiat, 1979).

*Lactococcus lactis* is commonly associated with dairy products (Song et al., 2017), and originated from plants. *L. lactis* can colonize stably in the intestinal tract because it has mucus/mucin binding proteins on the cell surface (Mercier-Bonin and Chapot-Chartier, 2017). Emerging evidence suggests that the colonization of *L. lactis* has an impact on gut microbiota and further on host health (Veiga et al., 2014; Derrien and van Hylckama Vlieg, 2015). For example, *L. lactis* was reported to be responsible for a significant increase in the number of *Bifidobacterium* cells in fecal samples (Bernbom et al., 2006). In the present study, the abundance of *Bifidobacterium pseudolongum* was found to be significantly higher in the CAD group, which could be induced by *L. lactis*.

Several natural *L. lactis* isolates have been shown beneficial for health (Mercier-Bonin and Chapot-Chartier, 2017; Nakano et al., 2018). Recombinant *L. lactis* has also been proposed as a delivery vehicle for therapeutic molecules in the gastrointestinal tract (Carvalho et al., 2017), due to its anti-inflammatory (Nishitani et al., 2009; Bermudez-Humaran et al., 2013; Luerce et al., 2014; Ballal et al., 2015), anti-cancer (Roeffen et al., 2015) and anti-diabetes properties (Freudenberg et al., 2013; Agarwal et al., 2014). Transcriptome analysis indicated that the casein diet upregulated PPAR signaling pathway, AMPK signaling pathway and lipolysis that are associated with obesity. In addition, PLS-DA analysis showed that the mRNA levels of Adipoq and Irs1 were correlated positively with the abundance of the core species *L. lactis*. The Adipoq gene encodes adiponectin, which modulates a number of metabolic processes, including glucose regulation and fatty acid oxidation (Díez and Iglesias, 2003). Irs1 encodes insulin receptor substrate−1, which is a substrate of the insulin receptor tyrosine kinase and plays a central role in the insulin-stimulated signal transduction pathway (Kovacs et al., 2003). The mRNA levels of these two genes were reported to be inversely correlated with body mass index, insulin resistance and the risk of type 2 diabetes (Hu et al., 1996; Carvalho et al., 1999; Ukkola and Santaniemi, 2002; Kursawe et al., 2010; Freudenberg et al., 2013). *L. lactis*, Adipoq and Irs1 have been shown negatively correlated with obesity development, however, we found strongly positive correlation among relevant variables and its underling mechanism needs to be further explored. Here, we come to the conclusion that the differentially expressed genes and the differentially represented bacterial taxon could regulate lipid metabolism more strongly by CAD than by CHPD, which may prevent host from metabolic diseases. In fact, casein has already been reported as an efficient protein source in preventing against obesity than meat proteins (Smith et al., 2015; Liisberg et al., 2016). This could be related to high levels of BCAAs in casein, including valine, leucine and isoleucine. Previous studies indicated that supplementation with BCAAs in diet increased the abundance of *Bifidobacterium* in gut, and some strains including *B. pseudolongum* may protect against diet-induced obesity (An et al., 2011; Moya-Pérez et al., 2015; Wang et al., 2015; Li et al., 2016). On the other hand, as recently shown for *Akkermansia muciniphila* (Ottman et al., 2017) and *Lactobacillus plantarum* (Heeney et al., 2019), an important route through which intestinal bacteria can benefit body health is by inducing improvements to intestinal epithelial barrier integrity, which could also be a clue for the mechanism exploration in the future.

## Conclusion

In conclusion, casein and CHPD have a substantial influence on the composition of gut bacteria and gene expression in cecum tissue. A short-term intervention with CAD induced a higher relative abundance of *L. Lactis*, *B. pseudolongum*, and higher mRNA levels of genes AdipoQ and Irs1 involved in obesity associated pathways compared with CHPD. Further work is required to explore the mechanism of how dietary proteins regulate gut microbiota and gene expression, and confirm the potential mechanism of obesity prevention.

## Data Availability Statement

The sequencing data are available on NCBI. The shotgun metagenomics sequencing accession ID is PRJNA545455. The transcriptome sequencing data accession ID is GSE131975.

## Ethics Statement

All animal experimental protocols were approved by the Animal Care Committee of Nanjing Agricultural University, and the animals were housed in an SPF animal facility (reference number SYXK(Su)2011-0037).

## Author Contributions

CL, GZ and XX designed the study. FZ and SS carried out the animal study and collected samples. YM conducted the RT-PCR verification experiment. FZ performed statistical analyses and produced the figures. FZ and CL wrote the manuscript.

## Conflict of Interest

The authors declare that the research was conducted in the absence of any commercial or financial relationships that could be construed as a potential conflict of interest.

## Footnotes

1 https://www.animalgenome.org/bioinfo/resources/manuals/SOAP.html

2 http://exon.gatech.edu/GeneMark/meta_gmhmmp.cgi

3 http://weizhong-lab.ucsd.edu/cdhit_suite/cgi-bin/index.cgi

4 http://www.genome.jp/kegg/

5 https://CRAN.R-project.org/package=mixOmics

## Supplementary Material

FIGURE S1 K-mer of metagenomic sequencing data.

FIGURE S2 Map of KEGG pathways. The colored orthorogy in the map represents the KOs existed in the result of current analysis. Red squares represent that the annotated KOs are more abundant in CHPD group, while green squares represent that in CAP groups. **(A)** Citrate cycle (TCA cycle). **(B)** Sulfur metabolism. **(C)** Galactose metabolism.

TABLE S1 **(A)** Growth performance of rats fed casein and chicken. **(B)** Ingredient composition and nutritional level of the diets. **(C)** Ingredient compositions of the mineral mixes for the two diets. **(D)** Amino acid compositions in the two diets.

TABLE S2 q-PCR Primer information.

TABLE S3 The basic information and useful rate of metagenomics data.

TABLE S4 Differential analysis based on DESeq2 at the genus and species level.

TABLE S5 Differential KOs of microbiota between the CAD and CHPD analysed by DESeq2.

TABLE S6 Defferential KEGG pathways of microbiota analysed by GSEA.

TABLE S7 The quality information of the cecal RNA-seq data.

TABLE S8 Differential analysis of cecal gene expression based on DESeq2.

TABLE S9 Differential KEGG pathway analysis of the cecum analysed by GSEA.

TABLE S10 Correlation matrix between cecal bacteria and the cecal gene expression.

## Abbreviations

CAD: casein-based diet
CHPD: chicken protein-based diet
KEGG: Kyoto Encyclopedia of genes and genomes.
