# Supplementary figures and images for: A Short-Term Feeding of Dietary Casein Increases Abundance of Lactococcus lactis and Upregulates Gene Expression Involving Obesity Prevention in Cecum of Young Rats Compared With Dietary Chicken Protein
Source: Front Microbiol. 2019 Oct 25;10:2411. doi: 10.3389/fmicb.2019.02411 (PMC6824296; doi:10.3389/fmicb.2019.02411)

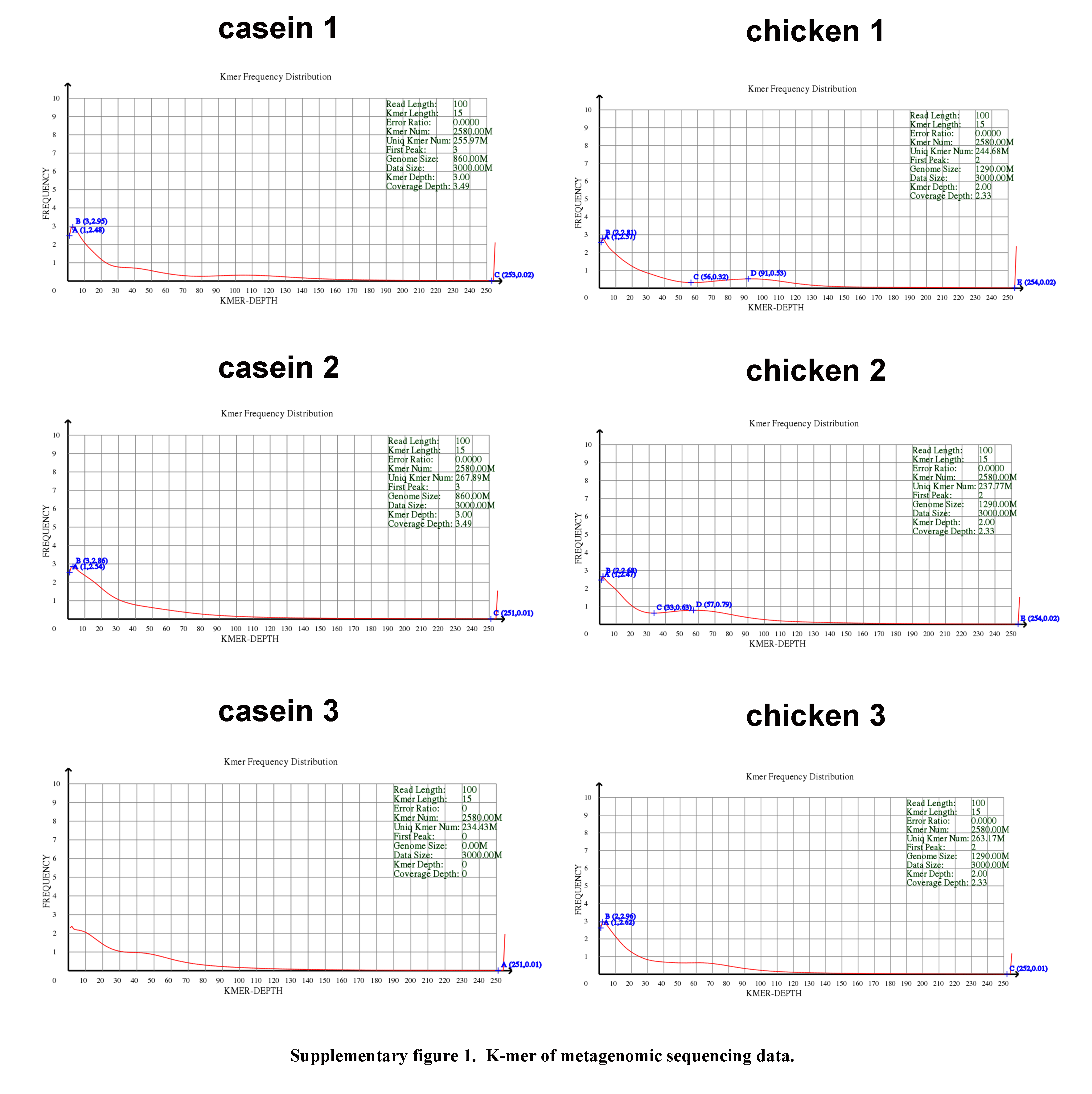

Supplement: FIGURE S1 — K-mer of metagenomic sequencing data. [file Image_1.TIF]

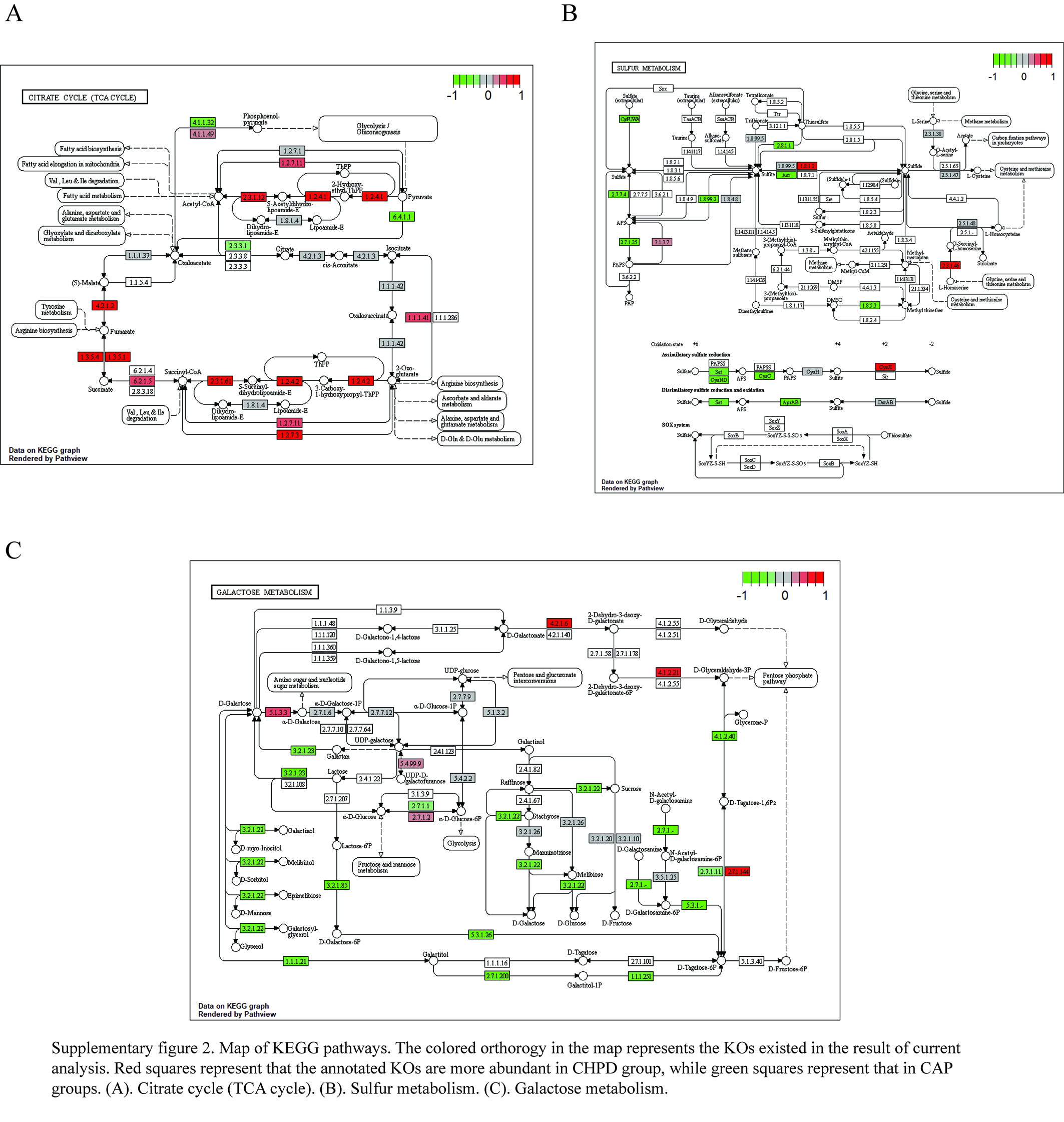

Supplement: FIGURE S2 — Map of KEGG pathways. The colored orthorogy in the map represents the KOs existed in the result of current analysis. Red squares represent that the annotated KOs are more abundant in CHPD group, while green squares represent that in CAP groups. (A) Citrate cycle (TCA cycle). (B) Sulfur metabolism. (C) Galactose metabolism. [file Image_2.TIF]
