# Supplementary material for: A Short-Term Feeding of Dietary Casein Increases Abundance of Lactococcus lactis and Upregulates Gene Expression Involving Obesity Prevention in Cecum of Young Rats Compared With Dietary Chicken Protein
Source: Front Microbiol. 2019 Oct 25;10:2411. doi: 10.3389/fmicb.2019.02411 (PMC6824296; doi:10.3389/fmicb.2019.02411)
Supplement: TABLE S1 — (A) Growth performance of rats fed casein and chicken. (B) Ingredient composition and nutritional level of the diets. (C) Ingredient compositions of the mineral mixes for the two diets. (D) Amino acid compositions in the two diets. [file Table_1.docx]

**Basic data of Growth performance and the diet**

**Data were quoted from the paper published previous by our group (Song et al, 2016)**

**Table 1A. Growth performance of rats fed casein and chicken**

|  | Casein | Chicken |
| --- | --- | --- |
| Initial BW, g | 87.91±5.46 | 90.77±3.69 |
| Final BW, g | 134.25±8.48 | 140.28±6.54 |
| BW gain, g/day | 7.72±0.64 | 8.25±0.77 |
| FI, g/day | 12.67±1.1 | 14.09±0.62* |

Values are mean ± SD; n = 10 per group; *: indicate significant difference (P < 0.05) compared to casein (reference) according to ANOVA with two-way Dunnett’s post-hoc test. BW: body weight; FI: feed intake.

**Table 1B.** **Ingredient composition and nutritional level of the diets**

| Items | Casein | Chicken | |
| --- | --- | --- | --- |
|  | **diet composition, g/kg diet** | |  |
| Purified protein^1^ | 200.0 | 192.0 | |
| Cornstarch | 397.5 | 397.5 | |
| Dyetros | 132 | 132 | |
| Sucrose | 100 | 100 | |
| Soybean oil | 70 | 70 | |
| Cellulose | 50 | 50 | |
| Mineral mix^2^ | 35.0 | 31.4 | |
| AIN-93 Vitamin mix | 10 | 10 | |
| L-Cystine^3^ | 3.0 | 0 | |
| Choline Bitartrate | 2.5 | 2.5 | |
|  | **nutritional level, units/kg** | |  |
| Energy，kcal | 4056.0 | 4056.0 | |
| Protein, g | 177 | 177 | |
| Fat，g | 70 | 70 | |
| Carbohydrate, g | 679.5 | 679.5 | |

Purified protein^1^, the amount of purified meat protein powder in the diet was adjusted and balanced according to the protein content in meat protein powders. Mineral mix^2^, the formulation of mineral mixes for six diets was listed in the **Table 3**. L-Cystine^3^: amino acid composition in diets were not modified.

**Table 1C. Ingredient compositions of the mineral mixes for the two diets**

| Ingredients, g/Kg | Casein | Chicken |
| --- | --- | --- |
| calcium carbonate, anhydrous, Ca 40.04% | 357.00 | 371.41 |
| potassium dihydrogen phosphate, P 22.76% K 28.73% | 196.00 | 182.48 |
| calcium phosphate, Ca 23.29% P 18.00% | 0.00 | 54.27 |
| potassium Citrate, K 36.16% | 70.78 | 0.00 |
| sodium chloride, Na 39.34% | 74.00 | 73.42 |
| potassium sulphate, S 18.39% K 44.87% | 46.60 | 51.91 |
| magnesium oxide, Mg 60.32% | 24.00 | 15.29 |
| iron citrate, Fe 16.5% | 6.06 | 0.00 |
| zinc carbonate, Zn 52.14% | 1.65 | 1.77 |
| manganous carbonate, Mn 47.79% | 0.63 | 0.71 |
| curpic carbonate, Cu 57.47% | 0.30 | 0.35 |
| potassium iodate, I 59.3% | 0.01 | 0.01 |
| sodium selenate, Se 41.79% | 0.01025 | 0.00000 |
| ammonium paramolybdate, 4 hydrate, Mo 54.34% | 0.00795 | 0.00886 |
| sodium meta-silicate, 9 hydrate, Si 9.88% | 1.45 | 1.62 |
| chromium potassium sulfate, 12 hydrate, Cr 10.42% | 0.275 | 0.306 |
| lithium chloride, Li 16.38% | 0.0174 | 0.0194 |
| boric acid, B 17.5% | 0.0815 | 0.0908 |
| sodium fluoride, F 45.24% | 0.0635 | 0.0707 |
| nickel carbonate, Ni 45% | 0.0318 | 0.0354 |
| ammonium vanadate, V 43.55% | 0.0066 | 0.0074 |
| sugar | 221.026 | 246.216 |
| total | 1000.00 | 1000.00 |

**Table 1D. Amino acid compositions in the two diets**

| Animo acids, g/Kg | Casein | Chicken | |
| --- | --- | --- | --- |
| asp | 1.42±0.02 | 1.79±0.04* | |
| thr | 0.87±0.01 | 0.87±0.02 | |
| ser | 1.17±0.01 | 0.77±0.02* | |
| glu | 4.85±0.09 | 3.15±0.06* | |
| gly | 0.37±0.01 | 0.75±0.01* | |
| ala | 0.62±0.01 | 1.09±0.02* |  |
| cys | 0.37±0.03 | 0.15±0.005* | |
| val | 1.24±0.03 | 0.89±0.01* | |
| met | 0.54±0.003 | 0.51±0.01* | |
| ile | 0.97±0.02 | 0.86±0.02* | |
| leu | 1.92±0.03 | 1.6±0.03* | |
| tyr | 0.8±0.01 | 0.41±0.01* | |
| phe | 1.01±0.01 | 0.77±0.01* | |
| lys | 1.57±0.02 | 1.66±0.03* | |
| his | 0.55±0.01 | 0.56±0.01 | |
| arg | 0.71±0.01 | 1.2±0.02* | |
| pro | 2.16±0.01 | 0.64±0.02* | |

Values are mean ± SD; n = 3 per group; *: indicate significant difference (P < 0.05) compared to casein (reference) according to ANOVA with two-way Dunnett’s post-hoc test.
