# Supplementary material for: A Short-Term Feeding of Dietary Casein Increases Abundance of Lactococcus lactis and Upregulates Gene Expression Involving Obesity Prevention in Cecum of Young Rats Compared With Dietary Chicken Protein
Source: Front Microbiol. 2019 Oct 25;10:2411. doi: 10.3389/fmicb.2019.02411 (PMC6824296; doi:10.3389/fmicb.2019.02411)
Supplement: TABLE S2 — q-PCR Primer information. [file Table_2.docx]

**Supplementary Table 2: q-PCR Primer information**

| Primer Name | Sequence 5’-3’ |
| --- | --- |
| Rat-AdipoQ-F | CCCAATGTTCCCATTCGCTT |
| Rat-AdipoQ-F | CCGGAATGTTGCAGTGGAAT |
| Rat-Irs1-F | AGCTGCATAATCGGGCAAAG |
| Rat-Irs1-R | TAACCTGCCAGACCTCCTTG |
| Rat-Cd36-F | ATCCTCTCCCTCTCTGGTGT |
| Rat-Cd36-R | AAGAGCTAGGCAGCATGGAA |
| Rat-Pde3b-F | TCACGGCCTTTCTGAGTGAT |
| Rat-Pde3b-R | AAGGTCCTCAGTCCTTCACG |
| Rat-GAPDH-F | CAAGGCTGAGAATGGGAAGC |
| Rat-GAPDH-R | GAAGACGCCAGTAGACTCCA |
